# Plant-based bioactives and oxidative stress in reproduction: anti-inflammatory and metabolic protection mechanisms

**DOI:** 10.3389/fnut.2025.1650347

**Published:** 2025-10-09

**Authors:** Xue Liu, Tongtong Zeng, Enfeng Zhang, Chengli Bin, Qun Liu, Kun Wu, Yiping Luo, Shaobin Wei

**Affiliations:** ^1^Department of Gynecology, Hospital of Chengdu University of Traditional Chinese Medicine, Chengdu, China; ^2^Affiliated Women and Children's Hospital, School of Medicine, UESTC Chengdu Women's and Children's Central Hospital, Chengdu, China

**Keywords:** food bioactive compounds, antioxidant active ingredients, phytochemicals, functional foods, anti-inflammation, reproductive health

## Abstract

Oxidative stress plays a central role in reproductive disorders, with food bioactive compounds offering therapeutic potential through their antioxidant properties. This review examines antioxidant active ingredients from plant-based foods and their protective mechanisms in reproductive system oxidative stress management. Key phytochemicals including polyphenols (flavonoids, phenolic acids such as curcumin, resveratrol, and EGCG), carotenoids (lycopene, lutein), and organosulfur compounds demonstrate potent free radical scavenging capacity, regulate antioxidant enzyme activity, and inhibit lipid peroxidation through Nrf2 pathway activation and NF-κB inhibition. These natural food ingredients provide anti-inflammatory effects and metabolic benefits including improved insulin sensitivity and mitochondrial protection. Clinical evidence shows lycopene supplementation (4–8 mg/day) improves sperm motility and reduces DNA fragmentation in male infertility, resveratrol (150 mg/day) enhances ovarian reserve markers in female fertility, and curcumin reduces inflammatory markers (IL-8, TNF-α) in endometriosis while improving assisted reproductive outcomes. However, poor bioavailability limits therapeutic efficacy, with most compounds showing < 10% absorption. Advanced delivery technologies, including nanoencapsulation (5–30 fold enhancement), phospholipid complexation, and formulation with absorption enhancers (e.g., piperine), can substantially improve the bioavailability of these compounds for functional foods and dietary supplements. Emerging single-cell and multi-omics approaches provide powerful tools to unravel tissue-specific mechanisms, while future progress also depends on establishing uniform dosage standards and conducting rigorous safety assessments to address potential pro-oxidant effects and long-term interactions. Given that infertility affects 17.5% of adults globally, food-derived antioxidant interventions represent accessible strategies for managing reproductive disorders, supporting the development of nutraceuticals and novel foods for reproductive health protection.

## 1 Introduction

Global reproductive health has emerged as a pressing concern, posing multifaceted challenges not only to individual wellbeing but also to public health systems and demographic stability. According to the World Health Organization, approximately 8%−12% of reproductive-age couples experience fertility issues, with recent data indicating that infertility affects nearly 17.5% of adults globally—an alarming and escalating global health challenge (1, 2).

The implications of fertility problems extend far beyond clinical diagnoses. Affected individuals frequently experience psychological distress, such as anxiety, depression, and diminished self-esteem (3, 4). Families may endure prolonged infertility treatments that are emotionally taxing and financially burdensome, especially in healthcare systems with limited insurance coverage (4, 5). On a societal level, declining fertility contributes to demographic challenges including accelerated population aging, workforce shortages, and intergenerational imbalance (6, 7). These overlapping burdens highlight an urgent need for accessible and effective reproductive health interventions.

Emerging evidence increasingly implicates environmental deterioration, lifestyle modifications, unbalanced diets, and chronic psychological stress as contributing factors to fertility impairments (8–10). Redox imbalance has been recognized as a central pathological nexus linking these diverse risk factors to reproductive impairments (9, 11). This state arises when the cellular balance between reactive oxygen/nitrogen species production and antioxidant defense is disrupted, leading to cumulative molecular damage (12).

The reproductive system is particularly vulnerable to reactive oxygen species (ROS)-induced stress due to its unique structural and metabolic characteristics. Sperm membranes are rich in polyunsaturated fatty acids that are readily oxidized, while oocyte maturation and fertilization demand high mitochondrial activity, increasing sensitivity to oxidative imbalance (13, 14). Substantial research has demonstrated that oxidative stress contributes to the pathogenesis of a wide array of reproductive disorders, including oligoasthenozoospermia, ovarian insufficiency, endometriosis, polycystic ovary syndrome, and prostatitis, as well as reproductive impairments associated with systemic metabolic conditions such as diabetes (13, 15, 16).

Conventional therapeutic approaches, including hormonal treatments and assisted reproductive technologies, remain the primary strategies for infertility management (17–19). However, these interventions are often limited by side effects, variable success rates, and accessibility challenges, particularly in resource-limited settings, highlighting the need for alternative or complementary approaches (20–22).

Plant-derived foods contain a variety of bioactive compounds with strong antioxidant capacities (23). These natural agents confer protection via multiple mechanisms: directly scavenging free radicals, enhancing endogenous antioxidant systems, modulating redox-sensitive signaling pathways, and preserving mitochondrial integrity (24–26). Compared to synthetic antioxidants, their multifunctional nature, better safety profile, and broader cellular targets offer distinct therapeutic advantages (24, 25).

In light of the growing fertility-related burden and the limitations of current treatment modalities, plant-based interventions present a compelling research focus. Herein, this review synthesizes current knowledge on the regulatory effects of bioactive components from plant-derived foods on oxidative imbalance in the reproductive system. We further explore their potential in mitigating inflammation-related and metabolism-associated reproductive disorders and examine translational strategies to enhance bioavailability and promote functional food development for reproductive health protection.

## 2 Molecular mechanisms of oxidative stress in the reproductive system

Oxidative stress constitutes a critical pathological axis linking environmental exposures, metabolic imbalance, and reproductive disorders. Clarifying the underlying molecular mechanisms is essential to understand how redox imbalance alters gamete integrity, hormonal regulation, and tissue homeostasis. This framework provides a foundation for interpreting both physiological processes and pathological outcomes in reproductive health.

### 2.1 Generation of ROS and reproductive vulnerability

Oxidative stress arises in reproductive tissues through a convergence of mitochondrial dysfunction, inflammatory activation, and environmental insults, with profound implications for gamete viability and hormonal regulation (27–29). Mitochondrial electron leakage during oxidative phosphorylation and NADPH oxidase activation serve as the primary endogenous sources of reactive oxygen species (ROS) in both male and female gonads (30, 31). Inflammatory leukocyte infiltration during ovulation and in the epididymal or seminal environment adds further ROS burden, especially under pathologic conditions (27, 29, 32). Exogenous contributors—including bisphenol A, heavy metals, ionizing radiation, and high-fat diets—amplify ROS generation or suppress antioxidant enzyme systems, tipping the redox balance toward cellular injury (33–36).

Notably, the structural composition of reproductive cells renders them uniquely vulnerable to oxidative damage: sperm membranes are rich in polyunsaturated fatty acids (PUFAs), which undergo rapid lipid peroxidation; spermatozoa also possess minimal cytoplasm, lacking significant antioxidant defense reservoirs (32, 37–41). Oocytes, while comparatively robust, contain a high density of metabolically active mitochondria and demand high ATP throughput, increasing both ROS production and mitochondrial stress under suboptimal conditions (31, 42, 43). Erectile tissues show similar vulnerability due to their dependence on nitric oxide (NO) signaling and high PUFA content in vascular smooth muscle membranes (44, 45). The corpus cavernosum contains high concentrations of polyunsaturated fatty acids in smooth muscle cell membranes, making them susceptible to lipid peroxidation. Additionally, the intricate vascular network required for erectile function depends on endothelial nitric oxide synthase (eNOS) activity, which is particularly sensitive to ROS-mediated inactivation and endothelial dysfunction.

The genomic and epigenomic integrity of gametes further raises the stakes: even subthreshold ROS-induced lesions may result in fertilization failure, impaired embryo development, or transgenerational genomic instability (42, 43, 46–48).

### 2.2 Physiological roles of ROS in reproduction

Far from being solely destructive, reactive oxygen species (ROS) at physiological concentrations are essential modulators of reproductive processes (49, 50). In males, ROS are involved in sperm capacitation through cholesterol efflux, membrane hyperpolarization, and tyrosine phosphorylation—prerequisites for acrosomal exocytosis and zona pellucida binding (49, 51, 52). In females, ROS facilitate follicular rupture, corpus luteum formation, and endometrial remodeling during the periovulatory phase, partly by enhancing matrix metalloproteinase activity and promoting local prostaglandin release (29, 53, 54). These coordinated redox changes act in tandem with inflammation-like signaling required for ovulation and implantation (53, 55). In erectile tissues, physiological ROS levels support normal vascular responses, but excess ROS rapidly inactivate nitric oxide, impairing erectile function (56).

**Figure 1:**
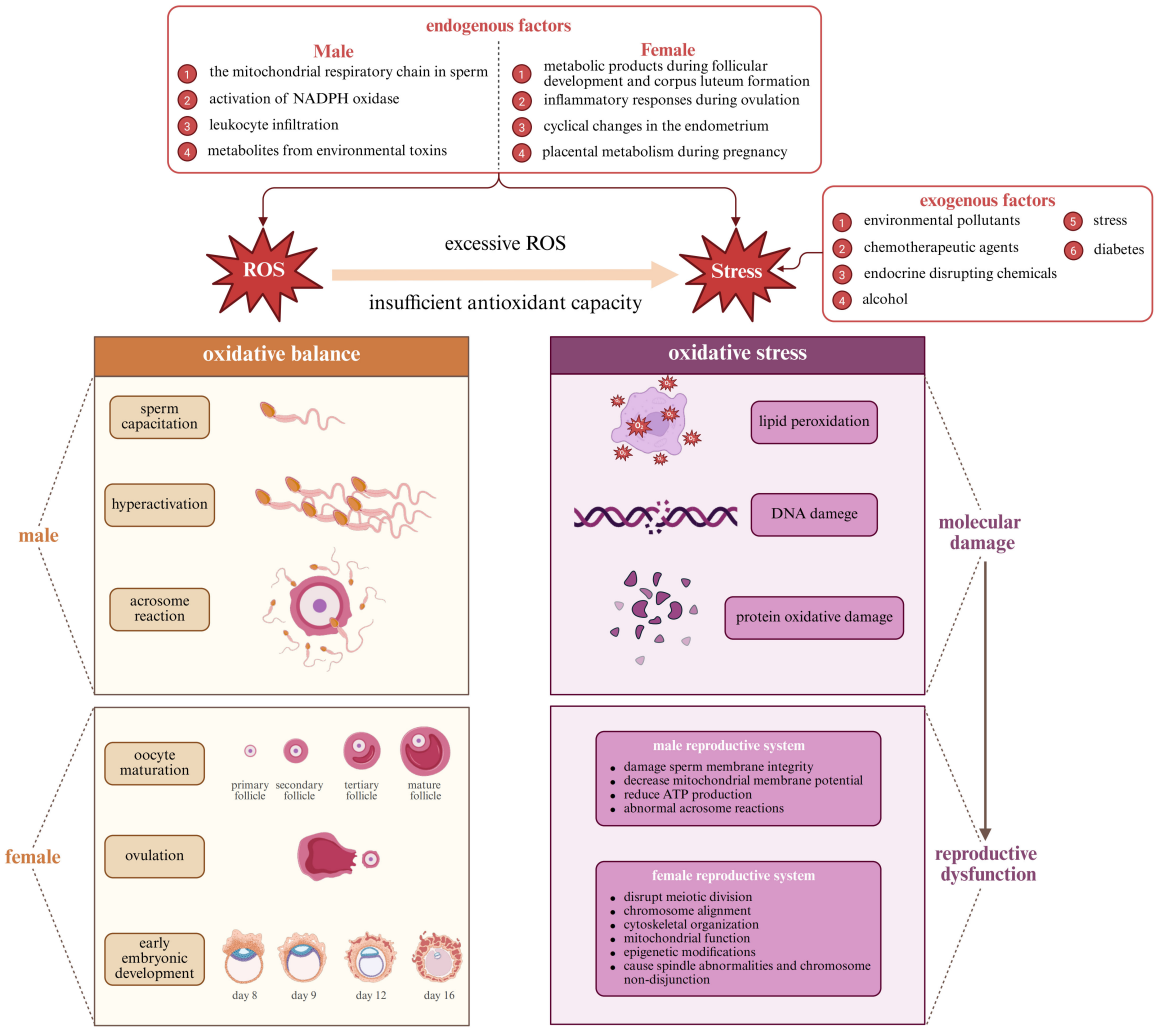
Antioxidant mechanisms of polyphenolic compounds (Created with BioRender.com).

Intracellularly, ROS serve as secondary messengers activating the MAPK (mitogen-activated protein kinase), PI3K/Akt (phosphoinositide 3-kinase/protein kinase B), and JNK (c-Jun N-terminal kinase) pathways, which regulate cytoskeletal remodeling, steroid biosynthesis, and controlled apoptosis (55, 57–59). The Keap1–Nrf2–ARE pathway (Kelch-like ECH-associated protein 1–Nuclear factor erythroid 2–Antioxidant Response Element), transiently activated during ovulation and implantation, induces antioxidant enzymes such as HO-1 (heme oxygenase-1) and NQO1 (NAD(P)H:quinone oxidoreductase 1), thereby providing cytoprotection without suppressing the physiological ROS signaling essential for fertilization and embryo development (60–64). The balance between beneficial and detrimental redox activity is depicted in Figure 1, which contextualizes ROS as both drivers and modulators of fertility-related cellular functions.

### 2.3 Oxidative stress-mediated reproductive dysfunction

Excessive accumulation of ROS disrupts reproductive function by impairing gamete integrity, altering hormonal signaling, and promoting chronic inflammation (65–67). In sperm, ROS-driven lipid peroxidation compromises membrane fluidity, reduces mitochondrial membrane potential, and elevates DNA fragmentation—all of which impair motility and fertilization capacity (32, 39, 68). Oocytes subjected to oxidative insult exhibit disrupted spindle microtubule assembly, chromosomal missegregation, and mitochondrial dysfunction, contributing to aneuploidy and embryo arrest (66, 69, 70). In parallel, ROS dysregulate the hypothalamic-pituitary-gonadal axis by inhibiting GnRH pulsatility, suppressing gonadotropin secretion, and impairing steroidogenic enzyme function in the gonads. Follicular atresia and testicular germ cell apoptosis are accelerated, thereby reducing ovarian reserve and sperm output (32, 39, 65, 68, 71). ROS also amplify inflammation by activating NF-κB, which induces cytokines such as IL-6 and TNF-α, creating a self-perpetuating inflammatory-oxidative feedback loop that degrades reproductive tissues over time (71–73). The cumulative impact of these mechanisms is diagrammed in Figure 1, highlighting the systemic nature of ROS-induced reproductive failure.

### 2.4 Oxidative damage biomarkers in the reproductive system

**Figure 2:**
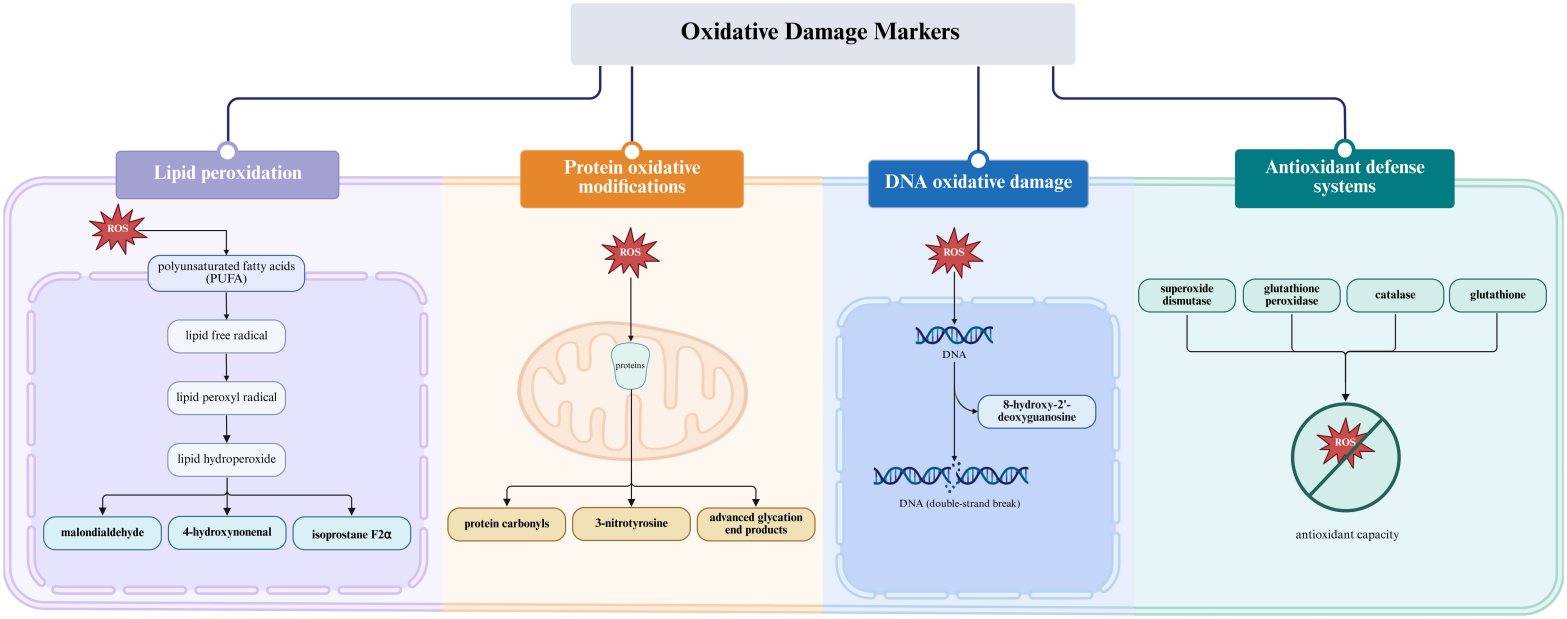
Oxidative balance and oxidative stress in reproductive systems (Created with BioRender.com).

Biochemical markers of oxidative damage provide important diagnostic and mechanistic insights into redox imbalance in reproductive biology (29, 74). Among these, lipid peroxidation indicators, such as malondialdehyde (MDA), 4-hydroxynonenal (4-HNE), and 8-isoprostane, are frequently used to assess oxidative damage in sperm and oocyte membranes (75–77). Protein oxidation products, including protein carbonyls and nitrated residues like 3-nitrotyrosine, can compromise enzymatic activity essential for gamete fusion and fertilization (77–82). The DNA oxidation marker 8-hydroxy-2′-deoxyguanosine (8-OHdG) is widely recognized as a surrogate indicator of ROS-mediated genotoxicity and has been associated with embryo loss and recurrent miscarriage in both natural conception and assisted reproductive technology (ART) settings (76, 83, 84). Impaired antioxidant defenses, characterized by reduced activity of superoxide dismutase (SOD), glutathione peroxidase (GPx), and catalase (CAT), along with decreased glutathione levels, are commonly observed in patients with polycystic ovary syndrome (PCOS), endometriosis, and idiopathic infertility (43, 77, 85–87). Figure 2 classifies these biomarkers based on their molecular origin and functional relevance, highlighting their value in assessing oxidative damage and monitoring therapeutic outcomes. Table 1 provides a comprehensive overview of these oxidative stress biomarkers, their tissue distribution, associated reproductive conditions, and clinical significance for diagnostic and therapeutic monitoring.

**Table 1:** Oxidative stress biomarkers in reproductive pathology.

**Biomarker category**	**Specific markers**	**Tissue/Sample**	**Associated conditions**	**Clinical significance**
Lipid peroxidation	MDA, 4-HNE, 8-isoprostane	Sperm membranes, oocyte membranes	Oligoasthenozoospermia, ovarian insufficiency	Membrane damage in PUFA-rich reproductive cells
Protein oxidation	Protein carbonyls, 3-nitrotyrosine	Seminal plasma, follicular fluid	Fertilization failure, embryo arrest	Compromised enzymatic activity for gamete fusion
DNA damage	8-OHdG	Sperm DNA, oocytes	Embryo loss, recurrent miscarriage	Genotoxicity in natural conception and ART
Antioxidant depletion	SOD, GPx, CAT activity; GSH levels	Serum, follicular fluid	PCOS, endometriosis, idiopathic infertility	Impaired antioxidant defenses

## 3 Major plant-derived food bioactive substances and their antioxidant properties

### 3.1 Polyphenols: chemical structure and antioxidant mechanisms

Polyphenolic compounds represent nature's most diverse and abundant antioxidants, characterized by their multiple phenolic hydroxyl groups attached to aromatic rings (88, 89). These plant secondary metabolites comprise several major structural classes including flavonoids (quercetin, kaempferol, and apigenin), catechins (epigallocatechin gallate, epicatechin), anthocyanins (cyanidin, delphinidin), and phenolic acids (caffeic acid, ferulic acid) (90–92). The antioxidant capacity of polyphenols correlates directly with their chemical structure, particularly the number and position of hydroxyl groups, presence of extended conjugation, and spatial configuration (92, 93).

Polyphenols exert antioxidant effects through multiple mechanisms beyond simple free radical neutralization. At the molecular level, their phenolic hydroxyl groups readily donate hydrogen atoms to neutralize reactive oxygen and nitrogen species, forming relatively stable phenoxyl radicals through resonance delocalization across aromatic rings (94–97). Additionally, many polyphenols effectively chelate transition metals such as iron and copper, preventing Fenton reactions that generate highly reactive hydroxyl radicals (94, 98–100).

Beyond direct chemical interactions with ROS, polyphenols modulate cellular signaling pathways that regulate oxidative homeostasis. A particularly significant mechanism involves activation of the Keap1-Nrf2 (Kelch-like ECH-associated protein 1-Nuclear factor erythroid 2) pathway (101, 102). Polyphenols modify Keap1 through covalent interactions or phosphorylation events, releasing Nrf2 from cytoplasmic sequestration (101, 102). Translocated to the nucleus, Nrf2 binds to Antioxidant Response Element (ARE) sequences in the promoter regions of numerous antioxidant enzymes, including glutathione S-transferase, NAD(P)H:quinone oxidoreductase 1, and heme oxygenase-1, effectively amplifying endogenous antioxidant capacity (103, 104).

**Figure 3:**
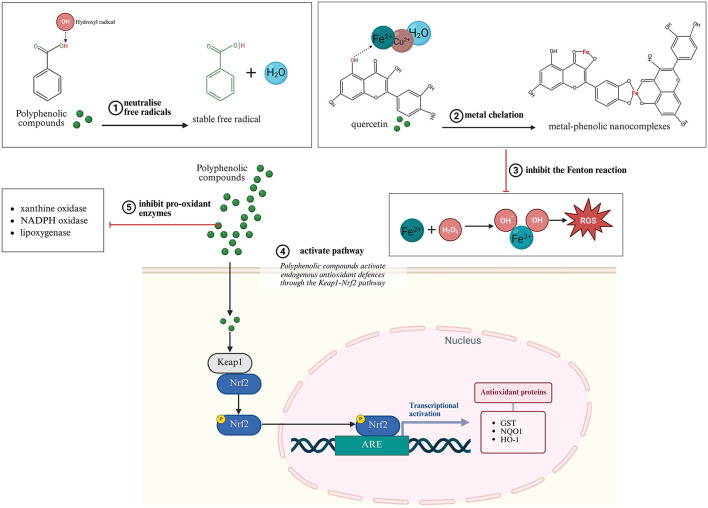
Biomarkers of oxidative stress (Created with BioRender.com).

Simultaneously, many polyphenols inhibit pro-oxidant enzymes like xanthine oxidase, NADPH oxidase, and lipoxygenase, further reducing ROS generation at its source (105–110). This multi-level intervention in oxidative processes contributes to their potent protective effects in reproductive tissues. These antioxidant mechanisms of polyphenolic compounds are illustrated in Figure 3.

### 3.2 Carotenoids: lycopene and lutein

**Figure 4:**
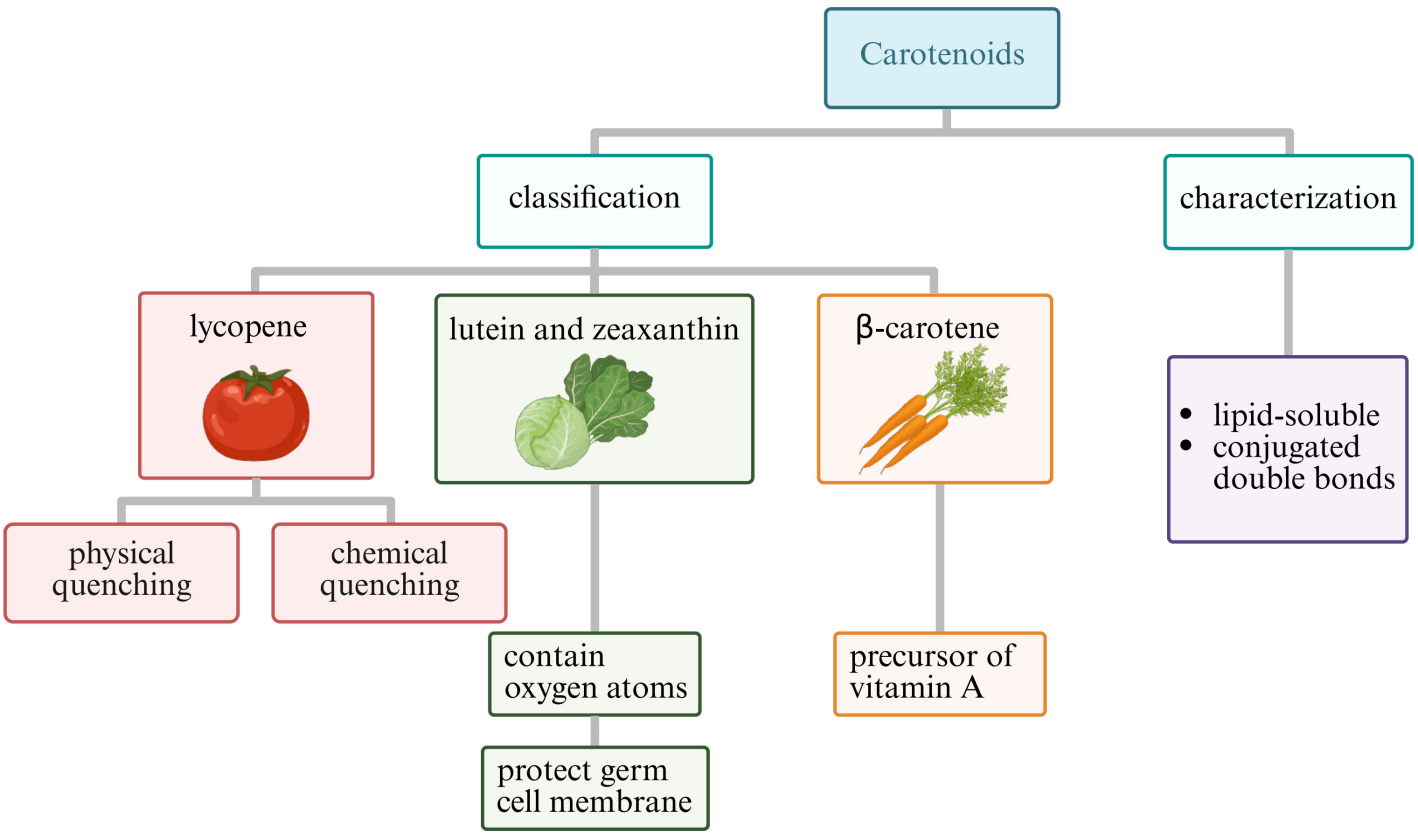
Classification of carotenoids relevant to reproductive health (Created with BioRender.com).

Carotenoids constitute a family of lipophilic pigments characterized by a polyisoprenoid structure with an extensive conjugated double bond system (111). This chemical architecture enables carotenoids to quench singlet oxygen and neutralize peroxyl radicals particularly efficiently, with their antioxidant activity correlating directly with the number of conjugated double bonds (112, 113). The most biologically relevant carotenoids for reproductive health include lycopene (predominant in tomatoes) and lutein (abundant in green leafy vegetables), and β-carotene (found in orange and yellow vegetables) (111–115). The structural classification and distinct functional properties of major reproductive-relevant carotenoids are illustrated in Figure 4.

Lycopene, containing 11 conjugated and two non-conjugated double bonds, demonstrates the highest singlet oxygen quenching capacity among common carotenoids—approximately twice that of β-carotene (116). Its acyclic structure contributes to its exceptional antioxidant properties (117). Particularly noteworthy is lycopene's tissue-specific accumulation pattern, with concentrations in the prostate gland reaching levels up to 10-fold higher than those in serum, suggesting specialized uptake mechanisms and particular relevance for male reproductive health (116, 118–121).

Mechanistically, lycopene functions through both physical and chemical quenching of reactive species (122–124). In physical quenching, the carotenoid absorbs energy from singlet oxygen, transitioning to an excited triplet state before dissipating the energy as heat, returning to ground state without chemical alteration (122, 123). This process can be repeated multiple times, allowing a single lycopene molecule to deactivate numerous singlet oxygen molecules (122, 124, 125). Chemical quenching involves electron transfer or addition reactions with free radicals, effectively terminating radical chain reactions but resulting in lycopene oxidation (126–128).

Lutein belongs to the xanthophyll subclass of carotenoids, distinguished by the presence of oxygen-containing functional groups. These polar groups affect their orientation within biological membranes, with lutein spanning the lipid bilayer perpendicular to the membrane surface (129, 130). This specific membrane organization enables lutein to efficiently intercept lipid peroxyl radicals before they initiate chain reactions, particularly protecting the polyunsaturated fatty acid-rich membranes of developing oocytes and sperm cells (131–133). Research indicates that lutein's membrane-stabilizing effects contribute significantly to maintaining mitochondrial integrity under oxidative challenge—a critical factor for energy-intensive reproductive processes (134).

Unlike some antioxidants with restricted tissue distribution, carotenoids effectively cross both the blood-testis and blood-follicle barriers, providing direct protection to gametes (135). Their strong lipophilicity also facilitates accumulation in steroidogenic tissues, where they protect steroid-synthesizing enzymes from oxidative damage, potentially preserving hormonal balance essential for reproductive function (136–138).

### 3.3 Other active components: organosulfur compounds

Beyond polyphenols and carotenoids, several other phytochemical classes demonstrate significant antioxidant activity relevant to reproductive health. Organosulfur compounds, predominantly found in Allium species (garlic, onions) and cruciferous vegetables, represent a structurally diverse group including allicin, diallyl sulfides, and isothiocyanates (139–141). These compounds feature reactive sulfur-containing functional groups that provide unique biochemical properties extending beyond conventional antioxidant mechanisms.

Allicin (diallyl thiosulfinate), formed when garlic is crushed through the enzymatic action of alliinase on alliin, contains a reactive thiosulfinate group that interacts with thiol-containing proteins. This interaction affects multiple redox-sensitive enzymes and transcription factors (142–144). Rather than acting primarily as direct radical scavengers, organosulfur compounds function as indirect antioxidants by potently inducing phase II detoxification enzymes through the Nrf2 pathway. Additionally, they upregulate thioredoxin and glutathione systems—critical components of cellular redox homeostasis in reproductive tissues (145–147).

S-allylcysteine and S-allylmercaptocysteine, water-soluble organosulfur derivatives found in aged garlic extracts, demonstrate particular efficacy in reproductive protection. These compounds preserve mitochondrial function under oxidative challenge, inhibit lipid peroxidation cascades, and modulate inflammatory prostaglandin production (148–151). Studies indicate they maintain sperm membrane integrity and motility when exposed to oxidative insults, suggesting specific applications in male fertility preservation (149, 150, 152).

### 3.4 Bioavailability and action targets

The therapeutic potential of plant-derived antioxidants faces a significant challenge: their limited bioavailability (91, 153, 154). The very chemical properties that make polyphenols and carotenoids such effective antioxidants—their aromatic rings, extensive conjugation, and hydroxyl groups—also contribute to poor water solubility, limited absorption, extensive first-pass metabolism, and rapid elimination. Most compounds demonstrate systemic bioavailability below 10% when administered in conventional forms, substantially limiting their biological effects (91, 153, 155–157).

Bioavailability varies considerably between compounds and depends on multiple factors including molecular size, lipophilicity, solubility, pKa, and matrix effects (158, 159). Carotenoids illustrate how food processing dramatically influences absorption—cooking tomatoes in oil increases lycopene bioavailability by up to fivefold compared to raw consumption, as heat disrupts cellular structures while lipids facilitate incorporation into mixed micelles necessary for intestinal uptake (158, 160, 161). For polyphenols like anthocyanins, intact glycosides are absorbed differently than their aglycone counterparts, with transporter-mediated uptake playing a crucial role (162, 163).

Compounds including curcumin and resveratrol face particularly profound bioavailability challenges. Despite demonstrated efficacy *in vitro*, curcumin's poor aqueous solubility, chemical instability at physiological pH, and extensive metabolism result in barely detectable plasma concentrations after oral administration (164–167). Similarly, resveratrol undergoes extensive sulfation and glucuronidation, with free resveratrol representing less than 1% of total plasma resveratrol after oral dosing (168–171).

To address these limitations, several innovative delivery strategies have emerged. Nanoencapsulation techniques using liposomes, solid lipid nanoparticles, or polymeric micelles dramatically improve water dispersibility while protecting compounds from premature degradation (172–174). Phospholipid complexation enhances membrane transport and tissue distribution by improving amphipathic properties (175). Formulation with absorption enhancers like piperine inhibits conjugating enzymes and efflux transporters, significantly increasing bioavailability. For instance, piperine co-administration increases curcumin bioavailability by up to 2,000%, though the clinical significance of this enhancement in humans requires further validation (176, 177).

The molecular targets of plant-derived antioxidants extend far beyond direct radical scavenging, revealing sophisticated mechanisms that explain their effects on reproductive health. Many compounds modulate key transcription factors that serve as master regulators of cellular redox status. Nuclear factor erythroid 2-related factor 2 (Nrf2) activation by polyphenols and curcumin triggers coordinated upregulation of dozens of cytoprotective enzymes, creating persistent protection that outlasts the compound's presence. Simultaneously, inhibition of nuclear factor kappa B (NF-κB) suppresses inflammatory cascades that would otherwise amplify oxidative damage (178–181).

Plant antioxidants also demonstrate remarkable specificity for critical reproductive targets. Epigallocatechin gallate and resveratrol modulate peroxisome proliferator-activated receptor gamma activity, improving insulin sensitivity crucial for hormonal balance in polycystic ovary syndrome (182–184). Flavonoids and carotenoids directly influence mitochondrial function—the energy and ROS production centers within reproductive cells—by stabilizing membranes, improving electron transport efficiency, and activating mitochondrial antioxidant systems. These targeted effects explain why plant-derived compounds often show reproductive benefits that exceed what would be predicted from their direct radical scavenging capacity alone (183). Table 2 summarizes the bioavailability challenges faced by major plant antioxidant classes and the corresponding enhancement strategies that have proven most effective in improving their therapeutic potential.

**Table 2:** Bioavailability enhancement strategies for plant-derived antioxidants.

**Compound class**	**Representative compound**	**Original bioavailability**	**Major limiting factors**	**Optimal enhancement strategy**	**Enhancement effect**	**Key clinical applications**
Flavonols	Quercetin	1–2%	Poor water solubility, extensive first-pass metabolism	Nano-liposome encapsulation	10–15 fold increase	Anti-inflammatory, cardiovascular protection
Catechins	EGCG	5–10%	Gastric acid degradation, protein binding	Nanoparticle delivery + vitamin C	3–5 fold increase	Neuroprotection, anticancer
Anthocyanins	Cyanidin	< 1%	pH sensitivity, rapid metabolism	Microencapsulation technology	5–8 fold increase	Vascular endothelial function improvement
Curcuminoids	Curcumin	< 1%	Extremely poor water solubility, rapid metabolism	Phospholipid complexes + piperine	20–30 fold increase	Anti-inflammatory, arthritis adjuvant therapy
Stilbenoids	Resveratrol	0.5–2%	Photosensitivity, glucuronidation	Lipid nanoparticles	5–10 fold increase	Anti-aging, cardiovascular protection
Carotenoids	Lycopene	10–30%	Heat/light instability, fat-dependent absorption	Microemulsification + isomerization	Enhanced to 50–70%	Prostate cancer prevention
Organosulfur compounds	Allicin	3–5%	High volatility, gastric acid decomposition	Enteric-coated formulation + stabilizers	3–4 fold increase	Antibacterial, antihypertensive

### 3.5 Integrated antioxidant mechanisms and signaling pathway regulation

Plant-derived antioxidants protect reproductive function through an integrated network of mechanisms that extend beyond simple ROS neutralization. The coordinated regulation of multiple signaling pathways generates synergistic protective effects, addressing the multifactorial nature of reproductive oxidative stress through the engagement of diverse cellular defense systems.

The Kelch-like ECH-associated protein 1–Nuclear factor erythroid 2–Antioxidant Response Element pathway (Keap1–Nrf2–ARE) is a master regulator of cellular antioxidant responses and a primary target for many plant compounds (185–187). Under basal conditions, Nrf2 remains sequestered in the cytoplasm by Keap1 (64, 186, 187). Plant antioxidants can modify cysteine residues in Keap1, causing conformational changes that release Nrf2 and allow its nuclear translocation (186–188). Once in the nucleus, Nrf2 binds to antioxidant response elements and activates cytoprotective genes such as glutathione synthase, thioredoxin reductase, and heme oxygenase-1(64). This cascade amplifies antioxidant defenses and enhances resilience against oxidative insults in reproductive tissues.

The nuclear factor kappa B pathway (NF-κB ) represents another crucial target. Aberrant NF-κB activation drives pro-inflammatory cytokine production that exacerbates oxidative stress in reproductive pathologies (189, 190). Plant compounds such as curcumin, resveratrol, and EGCG inhibit NF-κB signaling by preventing IκB phosphorylation, blocking nuclear translocation, and suppressing DNA binding activity (16, 181, 189, 191). These anti-inflammatory actions directly complement antioxidant defenses, disrupting the feed-forward loop between inflammation and oxidative stress that underpins many reproductive disorders (16, 192).

Mitochondrial protection is equally significant for reproductive cells with high energy demands. Plant antioxidants activate SIRT1 and PGC-1α to promote mitochondrial biogenesis (193, 194), enhance the activity of mitochondrial antioxidant enzymes (193), improve electron transport chain efficiency (194), and regulate mitochondrial membrane permeability (194). Such preservation of mitochondrial integrity ensures sustained ATP production and reduces ROS overgeneration, processes that are critical for oocyte maturation and sperm motility.

Finally, metabolic regulation provides an additional protective layer, particularly in conditions such as PCOS and diabetic erectile dysfunction (ED) (195, 196). Plant compounds activate AMPK signaling, enhance glucose utilization, and regulate lipid metabolism (195). By addressing systemic metabolic dysregulation, these actions indirectly alleviate reproductive oxidative stress while reinforcing direct antioxidant effects.

**Figure 5:**
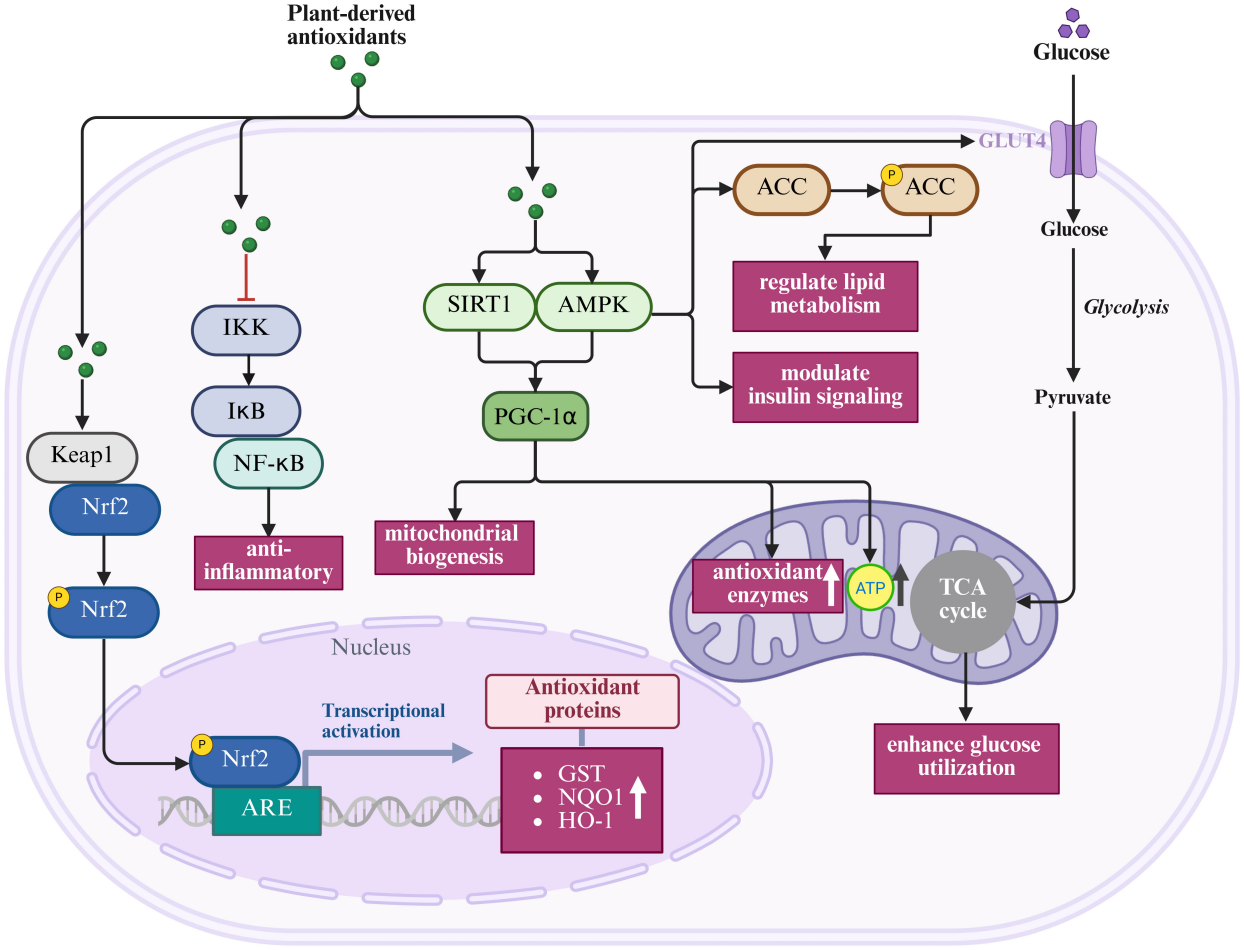
Integrated pathways regulated by plant-derived antioxidants (Created with BioRender.com).

Collectively, these mechanisms demonstrate how antioxidant, anti-inflammatory, mitochondrial, and metabolic pathways converge to safeguard reproductive health. Their simultaneous engagement distinguishes plant-derived antioxidants from conventional single-target drugs and helps explain their efficacy across diverse reproductive pathologies, as illustrated in Figure 5 and summarized in Table 3.

**Table 3:** Core regulatory pathways and synergistic mechanisms of major plant antioxidants in reproductive health.

**Active compound**	**Core regulatory pathway**	**Synergistic mechanism**
Curcumin	Keap1-Nrf2 + NF-κB inhibition	Inhibits NF-κB by blocking IκBα degradation while activating Nrf2 to enhance HO-1 expression
Resveratrol	SIRT1 activation → PGC-1α deacetylation → mitochondrial biogenesis	Combined with AMPK activation, promotes fatty acid oxidation and reduces ROS production
EGCG	Inhibits IKKβ phosphorylation (NF-κB pathway) + Fe^2^ + chelation (ROS neutralization)	Polyphenol hydroxyl structure simultaneously scavenges free radicals and regulates inflammatory gene transcription
Quercetin	Nrf2 nuclear translocation + inhibits MAPK/AP-1 inflammatory signaling	Flavonol structure enhances SOD activity, synergistically inhibits JNK/ERK phosphorylation
Lycopene	Physical quenching of singlet oxygen (ROS neutralization) + inhibits NF-κB nuclear translocation	Lipophilic properties protect membrane structure, reducing oxidative stress-induced inflammation

## 4 Plant antioxidant interventions in reproductive diseases: clinical evidence and therapeutic strategies

Plant antioxidant interventions have shown promising therapeutic potential across major reproductive disorders, with clinical evidence supporting their efficacy in treating fertility issues, inflammatory conditions, and metabolic dysfunction.

### 4.1 Interventions in fertility disorders

Oxidative stress represents a major contributing factor to both male and female infertility, making plant-derived antioxidants attractive therapeutic candidates with growing clinical evidence.

#### 4.1.1 Evidence-based approaches for male infertility

Oxidative stress represents a major contributing factor to male infertility, with studies indicating its involvement in 30%−80% of idiopathic infertility cases (197). Plant-derived antioxidants have demonstrated significant potential in addressing sperm oxidative damage and improving male fertility parameters through targeted interventions with strong clinical evidence (198–200).

Lycopene has emerged as one of the most well-studied plant antioxidants for male fertility. Human trials reported that lycopene supplementation (4–8 mg daily for 3–12 weeks) significantly improved sperm parameters (116). Mechanistically, lycopene's selective accumulation in the testes (reaching concentrations 10-fold higher than serum levels) enables direct protection of developing sperm cells from oxidative damage (201).

Coenzyme Q10, though not strictly a plant compound but available in various plant sources, has demonstrated consistent benefits in male infertility treatment (202, 203). A meta-analysis including eight randomized controlled trials with a total of 877 male participants showed that CoQ10 supplementation significantly increased total sperm count, sperm motility, and progressive motility, and also improved the rate of normal sperm morphology (203). These improvements correlate with decreased oxidative stress markers in seminal plasma and reduced sperm DNA fragmentation, confirming the antioxidant mechanism underlying the clinical benefits (204).

Green tea catechins represent another promising intervention. Studies have shown that the addition of green tea extract during sperm cryopreservation can significantly improve sperm motility and DNA integrity (205–207). The combined antioxidant and anti-inflammatory properties of green tea catechins appear particularly beneficial for men with elevated seminal inflammatory markers (208, 209).

Combination approaches may offer enhanced therapeutic potential compared to single-compound interventions. Evidence suggests that the combination of Serenoa repens (saw palmetto) with lycopene and selenium shows greater efficacy than Serenoa alone in reducing prostate inflammation. While this combination theoretically may improve sperm quality through multiple pathways, clinical studies are still needed to verify its specific effects on fertility parameters (210). This potential synergistic effect likely results from complementary mechanisms targeting different aspects of oxidative damage protection.

Practical clinical considerations for male infertility management include initiating antioxidant therapy for at least 3 months (corresponding to the spermatogenic cycle); higher doses may be required for men with severe oxidative stress or inflammatory conditions; regular monitoring of seminal oxidative stress markers to assess treatment response; combining plant antioxidants with lifestyle modifications for optimal results; and considering individualized approaches based on specific infertility factors.

#### 4.1.2 Botanical interventions for female fertility enhancement

Female reproductive function demonstrates particular vulnerability to oxidative damage, with both oocyte quality and ovarian reserve showing sensitivity to redox imbalances. The clinical application of plant antioxidants in female fertility has yielded promising results, though evidence levels vary across different compounds and conditions.

Resveratrol has shown remarkable potential in improving ovarian function and oocyte quality (211). In clinical research, resveratrol supplementation (150 mg/day for 3 months) significantly improved ovarian response in women with diminished ovarian reserve undergoing assisted reproduction, resulting in higher antral follicle counts and improved hormone profiles (212). The ability of resveratrol to activate SIRT1/FOXO3a pathways appears particularly beneficial for preserving follicular reserve and enhancing mitochondrial function in aging oocytes (213, 214).

Curcumin demonstrates significant potential for women with endometriosis-related infertility. A randomized clinical trial by Jannatifar et al. (215) investigated the effect of nanomicelle curcumin (120 mg/day for 10 weeks) in women with stage III/IV endometriosis undergoing assisted reproductive technology (ART). The study showed that nanomicelle curcumin supplementation significantly reduced inflammatory markers (IL-8 and TNF-α) and oxidative stress biomarkers (MDA) in follicular fluid, while increasing antioxidant enzyme levels (TAC, CAT, and SOD). These biochemical improvements translated to enhanced ART outcomes, including increased number of mature oocytes, improved fertilization rates, and higher quality embryos. This correlates with reduced oxidative stress biomarkers in follicular fluid and normalized inflammatory markers (215).

Clinical application guidelines for female fertility include individualized selection of plant antioxidants based on specific fertility issues (216); initiating treatment at least 3 months before conception attempts for optimal effect (217); regular monitoring of ovarian reserve markers to assess response; careful consideration of dosage, as excessive antioxidant supplementation may paradoxically impair normal reproductive processes by disrupting physiological ROS signaling essential for fertilization and embryo development; and special attention to formulation quality and bioavailability enhancement (183, 216). Additionally, the timing of intervention appears critical, with benefits maximized when treatment begins well before assisted reproductive procedures.

### 4.2 Plant antioxidants in inflammation-related reproductive system diseases

Inflammatory conditions of the reproductive system, particularly endometriosis and pelvic inflammatory disease (PID), represent significant clinical challenges with substantial oxidative stress components. Plant antioxidants have emerged as promising non-hormonal management options with favorable side effect profiles in these conditions.

Curcumin has demonstrated particular efficacy in endometriosis management (218, 219). The latest randomized, double-blind, placebo-controlled trial showed that nanocurcumin (80 mg/day) combined with dienogest for 8 weeks significantly improved dysmenorrhea, dyspareunia, chronic pelvic pain, and dyschezia in endometriosis patients, while enhancing quality of life and sexual function index (except for orgasm domain) (218). These clinical benefits correspond with curcumin's ability to inhibit endometriotic implant growth and invasiveness through multiple mechanisms, including inhibition of NF-κB activation, reduction of inflammatory cytokine production, decreased angiogenic factor expression, and induction of apoptosis in ectopic endometrial cells (219, 220). In endometriosis mouse models, curcumin treatment significantly reduced implanted endometrial lesions, attributed to inhibition of NF-κB translocation and reduction of angiogenic mediators (220).

Green tea polyphenols complement curcumin's effects in endometriosis management (221). Research indicates that EGCG can inhibit endometrial implant proliferation and adhesion while inducing apoptosis in ectopic tissue (221). Animal studies report that green tea extract reduced both the number and size of endometriosis lesions through antiangiogenic and antioxidant effects, suggesting effective disease progression control through simultaneous targeting of multiple pathological processes (221, 222).

Resveratrol represents another promising intervention, demonstrating powerful anti-inflammatory and antiangiogenic properties in endometriosis models (223). By inhibiting COX-2 and reducing prostaglandin synthesis, resveratrol mitigates inflammatory cytokine release. In an experiment, oral resveratrol administration to nude mice with human endometriosis implants significantly reduced lesion number and volume through blockade of NF-κB activation and disruption of the inflammatory microenvironment required for lesion maintenance (224).

For pelvic inflammatory disease, plant antioxidants serve as valuable adjuncts to antibiotic therapy (225). According to NHANES data, PID affects approximately 2.5 million women of reproductive age in the United States, and while antibiotic treatment can alleviate symptoms, poor obstetric outcomes and high recurrence rates persist. Studies indicate that antioxidant supplementation during and after antibiotic treatment can reduce residual oxidative damage, improve recovery rates, and potentially decrease the risk of post-inflammatory sequelae such as tubal factor infertility (226). This approach addresses the oxidative stress and inflammatory cascade that continue even after pathogen eradication. For example, a study on asiatic acid (AA) demonstrated that AA significantly inhibits oxidative stress, reduces cytokine and chemokine production, and decreases inflammatory cascade through inhibition of NLRP3 inflammasome and NF-κB pathway, similar to mechanisms observed with other plant antioxidants such as curcumin and resveratrol in suppressing NF-κB activation. Complementary and alternative medicine as an adjunctive therapy to Western medicine has shown significant efficacy in PID treatment through dual mechanisms of antioxidant and anti-inflammatory actions, providing new strategies for improving patient outcomes (227).

In male reproductive tract inflammation, particularly prostatitis and epididymitis, plant antioxidants have also shown promising therapeutic potentia. Chronic prostatitis, affecting up to 50% of men during their lifetime, presents significant therapeutic challenges with substantial oxidative stress components. Recent studies have demonstrated that plant-derived antioxidants effectively modulate inflammatory pathways in male reproductive tissues (228). For instance, lycopene has shown remarkable therapeutic effects in epididymitis through multiple mechanisms: significantly reducing inflammatory cytokines (IL-1β, IL-6, and TNF-α), enhancing antioxidant enzyme activity (SOD, GSH-PX, and CAT), and inhibiting the PI3K/AKT signaling pathway (229). Similarly, curcumin has demonstrated efficacy in prostatitis management by suppressing NF-κB activation and reducing pro-inflammatory mediators, while quercetin has been clinically proven to improve symptoms in chronic prostatitis patients (230). These plant-based compounds not only complement antibiotic therapy but also address the persistent oxidative stress and inflammatory cascade that continue after infection resolution, potentially reducing long-term complications such as infertility and chronic pelvic pain syndrome in male patients.

### 4.3 Plant antioxidants in metabolic reproductive disorders

Metabolic reproductive disorders such as PCOS and erectile dysfunction require comprehensive approaches that simultaneously address oxidative stress and underlying metabolic imbalances.

#### 4.3.1 Multi-target approach in polycystic ovary syndrome

Polycystic ovary syndrome (PCOS) represents a complex endocrine-metabolic disorder characterized by a vicious cycle between insulin resistance and oxidative stress (16, 231, 232). Plant antioxidants offer unique therapeutic potential through multi-target regulation of these interlinked pathological processes.

Cinnamon extract demonstrates remarkable efficacy in PCOS management (16). In a study of 80 women with PCOS, daily administration of 1,500 mg cinnamon powder capsules for 12 weeks significantly reduced fasting insulin and insulin resistance. Another double-blind randomized controlled trial showed that 3 g/day of cinnamon extract significantly decreased fasting blood glucose (*p* = 0.001) and glycosylated hemoglobin (*p* = 0.023) (16). These improvements are attributed to cinnamon's ability to enhance insulin receptor signaling and its polyphenols acting as insulin mimetics (233). Additionally, cinnamon's antioxidants alleviate systemic oxidative stress, evidenced by significantly reduced serum MDA levels in cinnamon-treated PCOS patients (234).

Green tea catechins similarly demonstrate capacity to improve PCOS metabolic and endocrine states. Clinical trials in overweight PCOS women found that green tea extract (rich in EGCG) 500 mg/day for 12 weeks led to significantly reduced free testosterone and fasting insulin levels compared to baseline (235). Decreased free testosterone indicates alleviated hyperandrogenemia, partly attributed to improved insulin sensitivity (235). Green tea polyphenols not only increase antioxidant defenses but also possess anti-androgenic effects; by reducing ovarian oxidative stress, EGCG may help restore more normal hormonal balance and promote ovulation (235).

Curcumin's anti-inflammatory and insulin-sensitizing effects effectively counter low-grade inflammation and metabolic dysfunction in PCOS (236, 237). In rodent models of PCOS, curcumin supplementation restored estrous cycles and reduced ovarian oxidative stress markers, improving oocyte quality and ovulation rates (238). Human research, though preliminary, shows encouraging results: PCOS patients taking curcumin (1,500 mg/day) for 12 weeks significantly lowered fasting blood glucose and insulin levels (237). Additionally, curcumin reduced oxidative stress biomarkers and increased total antioxidant capacity (236). These results align with curcumin's known ability to activate AMPK (enhancing insulin signaling) and upregulate Nrf2-dependent antioxidants, thereby breaking the insulin resistance-oxidative stress cycle (239).

Resveratrol also demonstrates significant metabolic and endocrine benefits in PCOS. In a double-blind trial, PCOS women taking resveratrol (1,500 mg/day) for 3 months showed 23% reduced total testosterone and 22% reduced dehydroepiandrosterone sulfate levels, while the placebo group showed no significant changes (240). This significant androgen level reduction suggests resveratrol directly improves ovarian steroidogenesis, possibly through reducing ovarian theca cell hyperresponsiveness. Resveratrol-treated patients also showed improved insulin sensitivity and mild weight reduction, though not all parameters reached statistical significance (240).

Clinical implementation considerations for PCOS include the importance of individualized approaches based on PCOS phenotype; the value of combining multiple plant antioxidants to address different aspects of this heterogeneous syndrome; and the need for sufficient treatment duration (minimum 12 weeks) to achieve measurable improvements in metabolic and reproductive parameters.

#### 4.3.2 Therapeutic strategies for erectile dysfunction

Erectile dysfunction (ED) represents a common complication with complex pathophysiology involving endothelial dysfunction and reduced nitric oxide (NO) bioavailability (241). Metabolic diseases such as diabetes, metabolic syndrome, and obesity contribute to ED through shared mechanisms (242–244). Plant antioxidants offer promising therapeutic options by targeting these fundamental mechanisms.

Panax Ginseng and its active components ginsenosides have attracted attention for their potential to improve erectile function (245, 246). Studies indicate that ginsenosides possess antioxidant properties that can enhance nitric oxide synthase (NOS) activity in cavernosal endothelial cells, reducing oxidative stress damage to vascular endothelium, thereby promoting NO-mediated smooth muscle relaxation and improving erectile function (245, 246). Systematic reviews of multiple clinical trials have shown that compared to placebo, ginseng preparations can significantly improve erectile function scores in ED patients, which is consistent with their antioxidant and NO-promoting mechanisms of action, despite some heterogeneity in study design and preparations used (247–249).

Pycnogenol^®^ (French maritime pine bark extract) is a standardized extract rich in powerful antioxidants including proanthocyanidins, catechins, and phenolic acids that directly target endothelial dysfunction associated with ED (250). Research confirms that Pycnogenol^®^ enhances endothelial NO production by increasing endothelial nitric oxide synthase (eNOS) activity and protects the generated NO from degradation by scavenging superoxide anion radicals, thereby improving its bioavailability (250). Its powerful antioxidant capacity helps reduce vascular oxidative stress. Clinical trials, particularly those combining Pycnogenol^®^ with the NO precursor L-arginine, report significant improvements in men's erectile function scores, an effect attributed to synergistically enhanced NO bioavailability and vascular endothelial protection (251).

Pomegranate (*Punica granatum*) is rich in potent polyphenolic antioxidants such as punicalagins and ellagic acid that effectively combat oxidative stress (252, 253). Preclinical studies and some clinical evidence suggest that pomegranate and its extracts can promote cardiovascular health by improving endothelial function and reducing oxidative stress levels (253, 254). Research indicates that pomegranate juice can enhance NO bioavailability by protecting NO from oxidative destruction and possibly upregulating eNOS expression (255). Although large-scale clinical evidence for ED is limited, a preliminary study observed improvements in erectile function scores in some men with mild to moderate ED after consuming pomegranate juice, suggesting the need for larger controlled trials for verification (256). The core mechanism for its potential vascular benefits (relevant to ED) is believed to be the reduction of systemic and vascular oxidative stress, thus protecting the NO signaling pathway critical for erectile response (253, 254).

Green tea catechins, particularly EGCG, show broad prospects for alleviating ED. In animal studies, EGCG supplementation preserved cavernosal smooth muscle content and improved erectile responses (257). One study found that rats receiving EGCG (with sildenafil) showed significantly increased eNOS expression and cyclic guanosine monophosphate levels in cavernosal tissues, with reduced MDA (lipid peroxidation marker) levels, compared to untreated groups (258). This indicates that EGCG enhances NO signaling and reduces oxidative damage in penile tissues (257). Researchers concluded that EGCG serves as a “cavernosal antioxidant,” potentially offering useful adjunctive therapy to PDE5 inhibitors for patients (258).

Curcumin, despite its broad antioxidant and anti-inflammatory effects, faces bioavailability challenges in ED treatment (259, 260). Recent innovations using topically applied curcumin-loaded nanoparticles found significantly improved erectile function parameters (261). This provides evidence that curcumin can protect erectile function by improving penile endothelial function and reducing fibrosis and oxidative damage. Studies have shown that administration of curcumin or its water-soluble conjugate led to enhancement of erectile function in diabetes induced-erectile dysfunction by stimulating increased synthesis of endothelial NOS and neuronal NOS (262, 263).

Clinical application considerations include the potential for plant antioxidants as adjunctive therapy alongside conventional PDE5 inhibitors; the importance of early intervention, ideally at the first signs of metabolic complications; and the value of addressing both metabolic control and oxidative stress simultaneously for optimal outcomes. Comprehensive treatment approaches combining lifestyle modifications with targeted antioxidant supplementation may provide the most effective strategy for improving erectile function, especially in cases where ED is driven by metabolic disorders and oxidative stress. Table 4 consolidates the clinical evidence for plant antioxidants across various reproductive disorders, including dosage regimens, primary outcomes, and underlying mechanisms demonstrated in human studies.

**Table 4:** Clinical evidence of plant antioxidants in reproductive disorders.

**Reproductive condition**	**Plant compound**	**Dosage/Duration**	**Primary outcome**	**Mechanism**	**Study type**
Male infertility	Lycopene	4–8 mg/day, 3–12 weeks	Improved sperm parameters	Selective testicular accumulation, membrane protection	Human trials
Diminished ovarian reserve	Resveratrol	150 mg/day, 3 months	Higher antral follicle counts, improved hormone profiles	SIRT1/FOXO3a activation	RCT
Endometriosis	Nanomicelle curcumin	120 mg/day, 10 weeks	Reduced inflammatory markers (IL-8, TNF-α), improved ART outcomes	NF-κB inhibition	RCT (Jannatifar et al.)
PCOS	Cinnamon powder	1,500 mg/day, 12 weeks	Reduced fasting insulin and insulin resistance	Insulin receptor signaling enhancement	Double-blind RCT
Erectile dysfunction	Panax Ginseng	Variable doses	Significant improvement in erectile function scores vs. placebo	NOS activity enhancement, oxidative stress reduction	Systematic reviews

## 5 Bioavailability enhancement strategies and functional food development

### 5.1 Formulation strategies for enhanced bioavailability

A key challenge limiting the clinical efficacy of plant antioxidants lies in their generally poor bioavailability. Novel formulation technologies have emerged to address this critical issue, significantly enhancing the therapeutic potential of these compounds in reproductive health applications.

Nanoencapsulation techniques represent a major advancement in plant antioxidant delivery (264, 265). Liposomal encapsulation markedly enhances the bioavailability of compounds like curcumin and resveratrol, with some studies reporting up to a five-fold increase in blood concentrations (266–268). Nanoemulsion technology can enhance the solubility and cellular uptake of lipophilic compounds such as lycopene and carotenoids, and may promote their distribution in biological fluids and improve *in vivo* bioavailability (269, 270). Solid lipid nanoparticles offer additional advantages of controlled release profiles and enhanced stability during gastrointestinal transit, which proves particularly valuable for compounds prone to degradation in acidic environments (271–273).

Phospholipid complexation substantially improves the pharmacokinetic profiles of many plant antioxidants (274, 275). Through the formation of amphipathic complexes with phospholipids, EGCG demonstrates enhanced membrane permeability and improved bioavailability (276). Animal pharmacokinetic studies have demonstrated that phospholipid-complexed curcumin exhibits approximately a fivefold increase in plasma concentration compared to standard curcumin formulations (277). This technology particularly benefits reproductive applications where penetration of blood-testis and blood-follicle barriers proves crucial for therapeutic efficacy.

Enzyme inhibition strategies represent another effective approach to enhancing bioavailability. Piperine, a major component of black pepper, inhibits UDP-glucuronosyltransferase and hepatic arylhydrocarbon hydroxylase, thereby reducing the first-pass metabolism of compounds such as curcumin. Clinical studies have demonstrated that co-administration of piperine (20 mg) with curcumin can increase curcumin's bioavailability by up to 2,000%, although this widely cited figure remains subject to debate regarding its actual therapeutic impact in clinical settings (278). Similarly, certain flavonoids like quercetin have been reported to inhibit metabolizing enzymes when co-administered with specific compounds, potentially prolonging their half-lives and enhancing therapeutic efficacy (279).

Chemical modification approaches, while more complex, offer significant potential. Developing water-soluble derivatives of lycopene and other carotenoids has shown promise in preclinical studies, with these modified compounds maintaining antioxidant activity while exhibiting superior absorption characteristics. Similarly, synthesizing pro-drug forms of plant polyphenols that undergo enzymatic activation in target tissues can enhance tissue-specific delivery and reduce systemic side effects.

Implementation considerations for clinical practice include recognizing that different plant antioxidants benefit from different enhancement technologies; considering potential interactions between delivery systems and the antioxidant mechanisms of the compounds; and acknowledging that enhanced bioavailability may necessitate dosage adjustments to maintain optimal safety profiles. Table 5 provides practical guidance on clinical dosing and bioavailability considerations for the major plant antioxidants discussed, facilitating evidence-based implementation in reproductive health practice.

**Table 5:** Clinical dosage and bioavailability considerations for plant antioxidants.

**Compound**	**Reported dosage in studies**	**Bioavailability challenge**	**Enhancement strategy**	**Clinical considerations**
Curcumin	Variable (clinical studies)	Extremely poor water solubility, rapid metabolism	Phospholipid complexes, piperine combination	Enhanced bioavailability may require dosage adjustment
Resveratrol	150 mg/day (ovarian reserve study)	Photosensitivity, glucuronidation	Lipid nanoparticles, quercetin synergy	Long-term compliance for reproductive benefits
EGCG	500 mg/day (green tea studies)	Gastric acid degradation, protein binding	Nanoparticle delivery, vitamin C combination	Timing relative to meals important
Lycopene	4–8 mg/day (male fertility studies)	Heat/light instability, fat-dependent absorption	Microemulsification, cooking with oil	Higher bioavailability with food processing
Cinnamon	1,500 mg/day (PCOS studies)	Not extensively discussed	Standardized extracts	Blood glucose monitoring in diabetic patients

### 5.2 Functional food development for reproductive health

Translating plant antioxidant research into accessible functional food products represents an important strategy for reproductive health protection. Functional food development requires specific design principles, with formulations incorporating complementary antioxidants in physiologically relevant ratios. For instance, combining lycopene, selenium, and zinc has demonstrated synergistic effects in improving male fertility (280). Matrix selection significantly impacts stability and bioavailability—lipid matrices enhance absorption of lipophilic antioxidants, while protein matrices support sustained release of polyphenolic compounds (281). Processing parameters must be optimized to maintain bioactivity while ensuring safety and shelf life, with techniques such as microencapsulation and freeze-drying widely applied (281). Sensory characteristics determine consumer acceptance, with taste and texture influencing long-term compliance—particularly important for reproductive interventions requiring extended periods (282, 283).

Fortified beverages represent widely applied functional food formats. Green tea beverages have demonstrated significant effects on male sperm parameters, including enhanced motility and DNA integrity protection (206, 284, 285). Pomegranate juice has shown improvements in mild erectile dysfunction, with mechanisms linked to enhanced nitric oxide bioavailability (286, 287). While convenient, beverages have limited carrier capacity for lipophilic compounds, which can be addressed through emulsifiers or nanoemulsion technologies (288–291).

Stability control represents a key challenge, addressed through co-antioxidants, microencapsulation techniques, and appropriate packaging systems (292–295). Quality control and standardization ensure safety and efficacy, including toxicological analysis and monitoring batch-to-batch variation. Regulatory frameworks vary between regions, with the US FDA allowing relatively relaxed structure-function claims, while EFSA requires health claims based on substantial clinical evidence (296–298).

In conclusion, functional food development for reproductive health has established systematic frameworks encompassing formulation design, carrier selection, and stability control. By optimizing these factors, the bioavailability and stability of plant antioxidants can be enhanced, providing practical pathways for application in reproductive health protection. Table 6 outlines potential functional food development applications based on the evidence presented, illustrating how plant antioxidants can be translated into practical interventions for reproductive health promotion.

**Table 6:** Functional food development applications for reproductive health based on evidence from research studies.

**Application area**	**Active compounds**	**Evidence from article**	**Development approach**	**Target benefits**
Male fertility support	Lycopene + CoQ10 + Green tea catechins	Clinical trials showing improved sperm parameters	Combination antioxidant formulations	Enhanced sperm motility and DNA integrity
Female reproductive health	Resveratrol + Curcumin	Studies on ovarian reserve and endometriosis	Targeted antioxidant interventions	Improved oocyte quality and reduced inflammation
Metabolic reproductive disorders	Cinnamon extract + Green tea polyphenols	PCOS studies with insulin sensitization	Standardized plant extracts	Glucose regulation and hormonal balance
General antioxidant beverages	Green tea catechins + Pomegranate polyphenols	Mentioned benefits for sperm DNA and erectile function	Natural beverage formulations	Daily antioxidant support for reproductive health
Enhanced bioavailability products	Various compounds with delivery systems	Extensive discussion of nano-encapsulation and phospholipid complexes	Advanced formulation technologies	Improved therapeutic efficacy

## 6 Future perspectives and conclusions

This review systematically explored the regulatory effects of bioactive components from plant-based foods on reproductive system oxidative stress and their protective mechanisms. By integrating the latest research advances, we examined the relationship between oxidative stress and reproductive dysfunction, analyzed how plant-derived antioxidants protect against inflammation-related and metabolism-related reproductive diseases, and evaluated their application in treating male and female infertility. From a food science perspective, we highlighted the sources, bioavailability, and optimal delivery methods of these bioactive compounds, providing a comprehensive framework for translating laboratory findings into practical dietary strategies and functional food development for reproductive health protection.

Future research should elucidate the tissue-specific mechanisms of plant active components in reproductive tissues; develop bioavailability enhancement technologies to overcome the limitations of low bioavailability; evaluate the synergistic effects of multiple plant active components to optimize combination strategies; and conduct standardized long-term clinical studies to establish optimal intervention protocols for different reproductive disorders. Emerging advanced techniques, such as single-cell RNA sequencing, spatial transcriptomics, and integrative metabolomics, offer powerful tools to address these questions. Single-cell and spatial approaches enable the dissection of tissue- and cell-type–specific gene expression patterns within reproductive organs, while high-resolution metabolomics combined with bioinformatics pipelines allows the identification of metabolic signatures that couple with these transcriptional programs (299). Such integrated multi-omics strategies will provide a concrete framework to link plant-derived antioxidant interventions with tissue-specific molecular pathways, thereby advancing precision reproductive medicine. Additionally, integration with functional genomics and bioinformatics will promote the development of personalized antioxidant intervention strategies.

Another critical future direction involves the establishment of uniform dosage standards for plant extracts in clinical trials. Variability in plant origin, cultivation conditions, harvest time, and processing methods often leads to inconsistencies in bioactive compound concentrations, thereby complicating dose–response evaluation. To overcome these uncertainties, standardized extraction protocols, chemical fingerprinting, and quantification of key bioactive components should be routinely applied. In addition, adherence to good manufacturing practice guidelines and the development of internationally recognized reference standards will be essential to ensure reproducibility and comparability across studies. Such measures will facilitate the reliable translation of plant-based bioactives into clinical and functional food applications.

It should also be noted that plant-derived antioxidants may exert dose-dependent biphasic effects, exhibiting potential pro-oxidant activity under specific concentrations or redox conditions. Moreover, prolonged or high-dose use could pose risks of unknown toxicity or adverse interactions, particularly when combined with pharmaceuticals or other dietary supplements. Therefore, future clinical research should incorporate rigorous dose–response studies, long-term safety evaluations, and systematic monitoring of potential drug–nutrient and nutrient–nutrient interactions to ensure both efficacy and safety in translational applications.

In addition, the future translation of these findings into clinical and functional food applications will require rigorous safety evaluation and compliance with regulatory frameworks to ensure both efficacy and consumer protection. Overall, plant-derived bioactive substances regulate reproductive system oxidative stress through multi-pathway protective mechanisms, providing significant interventions from inflammation inhibition to metabolic improvement. Combined with functional food development, these compounds hold strong potential to deliver safe, effective, and sustainable solutions for reproductive health challenges.
